# Supplementary material for: Eight quick tips for data-model integration in ecology
Source: PLoS Comput Biol. 2026 Jul 20;22(7):e1014524. doi: 10.1371/journal.pcbi.1014524 (PMC13384281; doi:10.1371/journal.pcbi.1014524)
Supplement: S1 Text — (PDF) [file pcbi.1014524.s001.pdf]

Supplemental Information

8 quick tips for data-model integration in ecology

Laurinne J Balstad[1,2, +, #,*], Joe Brennan[2,3,#,*], Marissa L. Baskett[1,2], Mattea K. Berglund[5], Mei Z. Blundell[1,2,4], Jessica A. Bolin[4,5], Amy A. Briggs[2,3], Mary C. Fisher[1,4], Christopher M. Heggerud[1,2,6], Madeline Jarvis-Cross[1,4], Lauren Mossman[1,4,7], Andrea N. Odell[1], Jennifer Paige[1,4,7], Sophia Pelletier[5], Mikaela M. Provost[5]

[1] Department of Environmental Science and Policy, University of California, Davis, California, United States of America;
[2] Center for Population Biology, University of California, Davis, California, United States of America;
[3] Department of Evolution and Ecology, University of California, Davis, California, United States of America;
[4] Coastal and Marine Sciences Institute, University of California, Davis, California, United States of America;
[5] Department of Wildlife, Fish, and Conservation Biology, University of California, Davis, California, United States of America;
[6] Department of Mathematics, University of Manitoba, Winnipeg, Manitoba, Canada;
[7] Department of Mathematics, University of California, Davis, California, United States of America
[#] Shared first authorship
[+] Present address: Department of Zoology, University of British Columbia, Vancouver, British Columbia, Canada
* Corresponding authors: Laurinne Balstad, laurinne.balstad@ubc.ca; Joe Brennan, jlbrennan@ucdavis.edu

Glossary A

Co-production of knowledge The process of jointly designing and implementing research projects with project partners, recognizing that project partners have the option to engage fully or in part at any step of the research process and equal authority over if or how data and findings are shared (S1).

Epistemology The philosophy of how and what we know and where that knowledge comes from (S2).

Generality How broadly applicable a model is across systems (S3).

Indigenous ecological knowledge (knowledge holders) “A cumulative body of knowledge, practice and belief evolving by adaptive processes and handed down through generations by cultural transmission, about the relationship of living beings (including humans) with one another and with their environment” ((S4), p. 7). Indigenous knowledge is unique to a people and place, and is “situated knowledge” in that it “is not separable from the knowledge holders/keepers or the environment in which it is embedded” ((S5), p. 246; (S6,S7))

Irreducible Uncertainty Uncertainty endogenous to the system, e.g., natural variability or inherent randomness (S8). *Reducible Uncertainty* arises from uncertainty not endogenous to the system, e.g., measurement or systematic error (S8).

Local ecological knowledge (knowledge holders) A “cumulative body of intergenerational knowledge, practices, values, and worldviews, and embedded in the relationships between local people and nature” ((S9), p. 483).

Precision The repeatability of a measurement or, for Levins’ triangle (S3), how testable a model’s outputs are. *Imprecision* can arise when there are conflicting or variable measurements.

Qualitative data Data that is not numeric, including narrative or descriptive data.

Quantitative data Data that is numeric rather than descriptive.

Realism (realistic) How accurately a model integrates the mechanisms of the observed biological system.

Sensitivity Analysis Approach to quantify variability of model output that can be attributed to uncertainty in specific input factors (S10–S12).
Uncertainty Analysis Approach to evaluate the overall variability in model output based on all uncertain inputs, generally with the goal of quantifying error around model predictions (S12–S15).

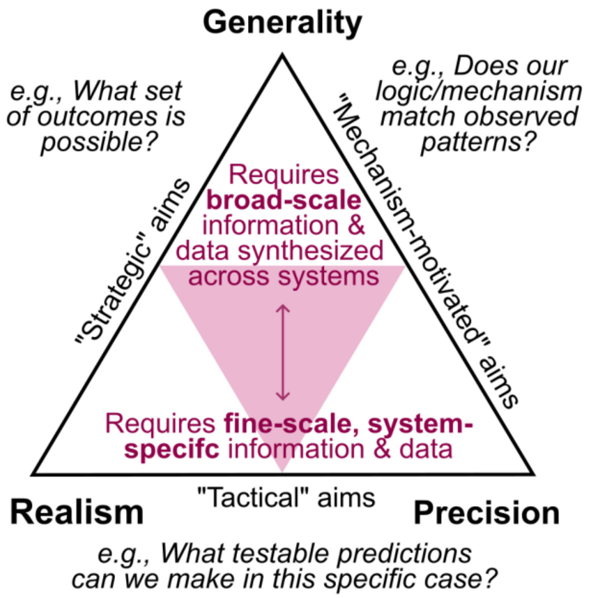

Figure A. Framing research question on the precision-generality-realism triangle, adapted from Levins (S3), in light of data and its uses. The kinds of questions a modeler asks are in black on the edges. The types of data that support addressing those aims are in pink in the center of the triangle; the horizontal width of the triangle represents the broadness of the data, where the narrow point at the bottom represents system- or case-specific information.

Table A: A non-exhaustive sample of how various data can be used in ecological modeling. For each row, we provide general quantitative and qualitative use examples and, in italics, a specific example of a theoretical ecology paper that draws on that data from either a quantitative or qualitative perspective. The data in each row can be found across a variety of peer-reviewed and grey literature sources. We categorize the data types broadly by row, acknowledging that there are overlaps in data types and these are not mutually exclusive types of data. *We define observational data as field observations of ecological or evolutionary processes with the goal of answering a specific scientific question, whereas we define natural history as observations of an ecological or evolutionary system that is agnostic to a specific scientific question and hence has a different objective than observational data.

**Data**	**Example quantitative use**	**Example qualitative use**
Observational data*	Time series, demographic rates, species interaction rates, genetic data	Synthesis of patterns from multiple field studies, qualitative trends identified from studies, descriptive

	Ex: Using mark-recapture data to parameterize a demographic model of tortoise subpopulation (S16)	observations<br><br>*Ex: Using conflicting qualitative patterns of disease occurrence in migrating species to motivate an ecological question* (S17)
Experimental data	Experimental time series, demographic rates, species interaction strength, genetic data<br><br>*Ex: Testing competing model mechanisms of anemone-symbiont relationships by parameterizing model to experimental data of anemone growth* (S18)	Mechanisms identified from experimental studies, descriptive observations<br><br>*Ex: Drawing on experimental observations to motivate a competition-colonization framework in a model of coral-symbiont dynamics* (S19)
Past theoretical literature	Past dynamical model parameters, past dynamical model output<br><br>*Ex: Using the same parameter set from a previous study to study different questions, allowing for synthesis across studies* (S20)	Patterns/mechanisms observed by past dynamical modelers, past model structures<br><br>*Ex: Using functional form from a previous study showing fear of predation in tri-trophic system to inform model structure* (S21)
Predictive or statistical model outputs as input data	Regional oceanic modeling system, climate models, inferred parameter estimates from past statistical models<br><br>*Ex: Using an oceanographic model output to estimate a connectivity matrix for larval dispersal in a spatially-explicit coral model* (S22)	Statistical models of community structure or interaction web<br><br>*e.g., The use of model-derived qualitative interaction webs to guide model structure, as done in some models of natural resource management* (S23)*, indicates potential for similar modeling approaches in theoretical ecology*
Expert opinion	Quantitative estimates of parameter values<br><br>*Ex: Using taxon-specific expert opinion to determine a quasi-extinction threshold for a population viability analysis* (S24)	Descriptive mechanisms, functional forms, population structure, species interactions, relevant outcomes, verbal or conceptual models of a system<br><br>*Ex: Partnering with industry members*

		to identify the functional form of parasite burden-host growth relationship in aquaculture system (S25)
Natural history*	Phenology time series, body size measurements, frequency of species interactions<br><br>*e.g., The existence of statistical analysis of natural history data, such as Thurough's recordings of phenology compared to modern-day recordings* (S26) *indicates the potential to use such natural history data to quantitatively parameterize dynamical models*	Descriptive field notes, historical observations, natural history letters<br><br>*Ex: Using past natural history observations of species hybridization* (S27) *to inspire a theory model of introgression leading to evolutionary rescue* (S28)
Mental models		Model goal, verbal models of population structure or species interactions<br><br>*Ex: Using mental model relating predation and sexual selection to mathematically model how they interact to shape dynamics of sexual displays* (S29)

<u>*Supplemental References*</u>

S1. Grimm J, Jarvis-Cross M, Bailey M, Ban NC, Bartlett M, Cadman R, et al. Co-producing knowledge with Indigenous Peoples: challenges and solutions. Trends Ecol Evol. 2025; 3533.

S2. Moon K, Cvitanovic C, Blackman DA, Scales IR, Browne NK. Five Questions to understand epistemology and its influence on integrative marine research. Front Mar Sci. 2021;8.

S3. Levins R. The strategy of model building in population biology. Am Sci. 1966;54(4):421–31.

S4. Berkes F. Sacred ecology. 4th ed. New York: Routledge; 2017. 394 p.

S5. Reid AJ, Eckert LE, Lane J, Young N, Hinch SG, Darimont CT, et al. "Two-Eyed Seeing": An Indigenous framework to transform fisheries research and management. Fish Fish. 2021;22(2):243–61.

S6. McGregor D. Coming full circle: Indigenous knowledge, environment, and our future. Am Indian Q. 2004;28(3):385–410.

S7. Prabhakar A, Mallory B. Guidance for Federal Departments and Agencies on Indigenous Knowledge. 2022.

S8. Regan HM, Colyvan M, Burgman MA. A taxonomy and treatment of uncertainty for ecology and conservation biology. Ecol Appl. 2002;12(2):618–28.

S9. IPBES. Global assessment report on biodiversity and ecosystem services of the Intergovernmental Science-Policy Platform on Biodiversity and Ecosystem Services. Brondizio ES, editor. IPBES secretariat, Bonn, Germany; 2019. 56p.

S10. Cariboni J, Gatelli D, Liska R, Saltelli A. The role of sensitivity analysis in ecological modelling. Ecol Model. 2007;203(1–2):167–82.

S11. Pianosi F, Beven K, Freer J, Hall JW, Rougier J, Stephenson DB, et al. Sensitivity analysis of environmental models: A systematic review with practical workflow. Environ Model Softw. 2016;79:214–32.

S12. Saltelli A, Tarantola S, Campolongo F, Ratto M. Sensitivity analysis in practice: A guide to assessing scientific models. John Wiley & Sons; 2004. 234 p.

S13. Loucks DP, van Beek E. System sensitivity and uncertainty analysis. In: Loucks DP, van Beek E, editors. Water Resource Systems Planning and Management: An Introduction to Methods, Models, and Applications. Cham: Springer International Publishing; 2017. p. 331–74.

S14. Malchow AK, Hartig F. Calibration, sensitivity and uncertainty analysis of ecological models--a review. Authorea [Preprint], 2025. Authorea [posted 2024 Nov 6]. Available from: https://www.authorea.com/doi/full/10.22541/au.173090741.12160653 doi: 10.22541/au.173090741.12160653/v1

S15. Saltelli A, Aleksankina K, Becker W, Fennell P, Ferretti F, Holst N, et al. Why so many published sensitivity analyses are false: A systematic review of sensitivity analysis practices. Environ Model Softw. 2019;114:29–39.

S16. Folt B, Goessling JM, Tucker A, Guyer C, Hermann S, Shelton-Nix E, et al. Contrasting patterns of demography and population viability among Gopher Tortoise populations in Alabama. J Wildl Manag. 2021;85(4):617–30.

S17. Shaw AK, Sherman J, Barker FK, Zuk M. Metrics matter: The effect of parasite richness, intensity and prevalence on the evolution of host migration. Proc R Soc B Biol Sci. 2018;285(1891):20182147.

S18. Kaare-Rasmussen JO, Moeller HV, Pfab F. Modeling food dependent symbiosis in Exaiptasia pallida. Ecol Model. 2023;481:110325.

S19. Gibbs TL, Dahlin KJM, Brennan J, Silveira CB, McManus LC. Coexistence of bacteria with a competition-colonization tradeoff on a dynamic coral host. bioRxiv [Preprint]. 2024 bioRxiv [posted 2024 Sept 16]. Available from:

https://www.biorxiv.org/content/10.1101/2024.09.15.612558v1 doi: 10.1101/2024.09.15.612558

S20. Shoemaker LG, Barner AK, Bittleston LS, Teufel AI. Quantifying the relative importance of variation in predation and the environment for species coexistence. Ecol Lett. 2020;23(6):939–50.

S21. Arroyo-Esquivel J, Adams R, Gravem S, Whippo R, Randell Z, Hodin J, et al. Multiple resiliency metrics reveal complementary drivers of ecosystem persistence: An application to kelp forest systems. Ecology. 2024;105(12).

S22. Vogt-Vincent NS, Pringle JM, Cornwall CE, McManus LC. Anthropogenic climate change will likely outpace coral range expansion. Sci Adv. 2025;11(23):eadr2545.

S23. Mehryar S, Sliuzas R, Schwarz N, Sharifi A, Van Maarseveen M. From individual Fuzzy Cognitive Maps to Agent Based Models: Modeling multi-factorial and multi-stakeholder decision-making for water scarcity. J Environ Manage. 2019;250:109482.

S24. Gregory KM, Darst C, Lantz SM, Powelson K, Ashton D, Fisher R, et al. Population viability analysis for two species of imperiled freshwater turtles. Chelonian Conserv Biol. 2024;23(1).

S25. Kragesteen TJ, Simonsen K, Visser AW, Andersen KH. Optimal salmon lice treatment threshold and tragedy of the commons in salmon farm networks. Aquaculture. 2019;512:734329.

S26. Miller-Rushing AJ, Primack RB. Global warming and flowering times in Thoreau's Concord: A community perspective. Ecology. 2008;89(2):332–41.

S27. Grant PR, Grant BR. Unpredictable Evolution in a 30-Year Study of Darwin's Finches. Science. 2002;296(5568):707–11.

S28. Baskett ML, Gomulkiewicz R. Introgressive hybridization as a mechanism for species rescue. Theor Ecol. 2011;4(2):223–39.

S29. Lerch BA, Servedio MR. Predation drives complex eco-evolutionary dynamics in sexually selected traits. PLOS Biol. 2023;21(4):e3002059.
